# 2115. Long Term Follow-up and Correlates of Success in Patients on Immune Globulin for Antibody Deficiency

**DOI:** 10.1093/ofid/ofac492.1736

**Published:** 2022-12-15

**Authors:** Olivia Duffield, Peter Axelrod

**Affiliations:** Temple University School of Medicine, Philadelphia, Pennsylvania; Temple University School of Medicine, Philadelphia, Pennsylvania

## Abstract

**Background:**

Antibody deficiencies range from mild deficits of antibody subclasses to more severe deficiencies of all immune globulins. Patients with deficiencies of immune globulin G (IgG) are predisposed to recurrent sinopulmonary infections, often necessitating multiple courses of antibiotics and frequent hospitalizations and provider visits. Treatment with intravenous immune globulin (IVIG) replacement therapy is currently indicated for patients with severe primary immune deficiencies but is less well studied in patients with more mild deficits that nonetheless experience recurrent infections.

**Methods:**

A retrospective chart review was conducted on 71 patients from the Temple University Infectious Disease Department (15 transplant recipients) with varying levels of antibody deficiency that had been treated with monthly IVIG for at least six months. Available patients (N=49) were interviewed by phone about their experiences on IVIG, side effects, and quality of life changes on therapy.

**Results:**

Mean age sx onset was 40 y,68% were women, 87% Caucasian, 49% had pneumonia, 65% purulent bronchitis, 65% sinusitis. Patients reported a mean reduction in infection frequency of 78% and average patient-reported improvement in quality of life was 4.7/5.0. Antibody deficiency levels at baseline were not correlated with infection reduction (R=-.05, p=0.71), Figure 1 and neither was response to pneumococcal polysaccharide vaccine (vaccine responder median infection reduction 83%; non responder 87%, p= 0.60) Figure 2. Adverse events on treatment were experienced by 59% of patients and caused 9 patients (13%) to stop treatment. There was no difference in incidence of adverse events between men and women (p= 0.076), but women had a 50% higher rates of severe headache than men (p= 0.038); 10% pts required a port or PICC, 32% IV fluids, 48% IV steroids, 31% IVIG brand change; 17% changed to subcutaneous IG.

**Figure 1:**
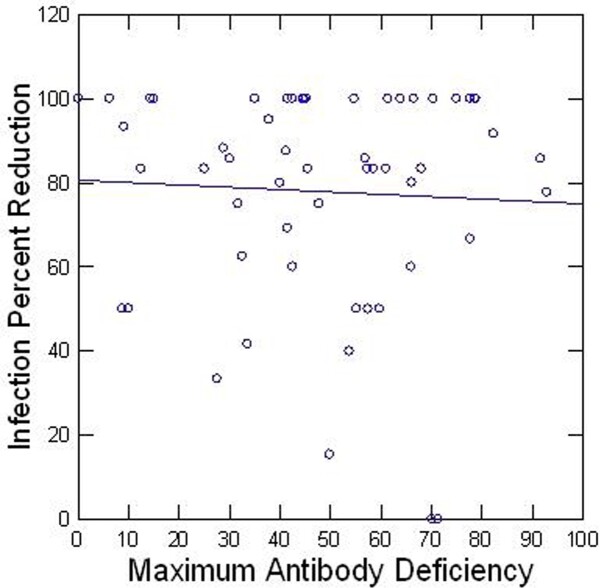
Pre-treatment Antibody Deficit and Infection Reduction by IVIG

**Figure:**
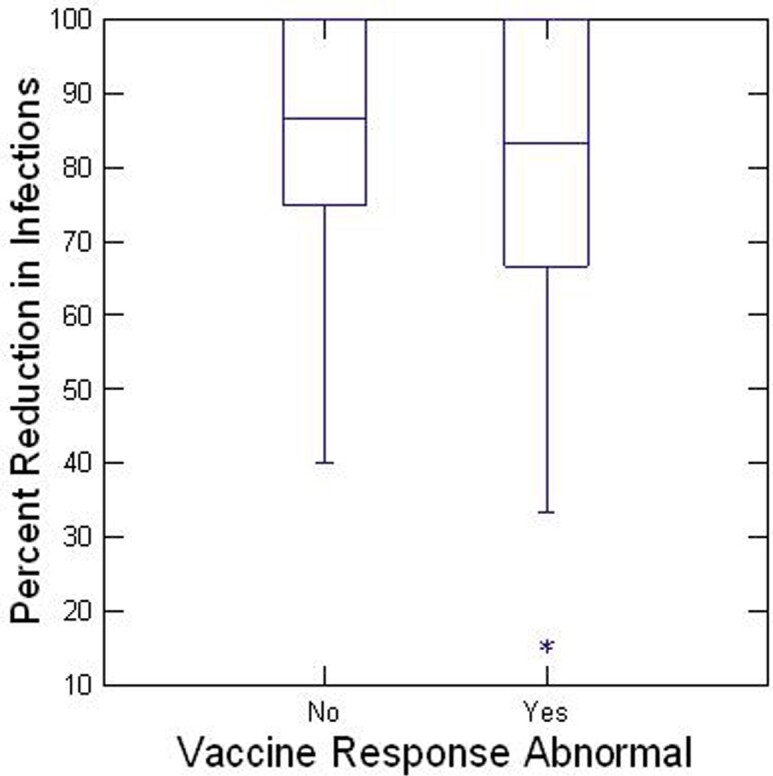

Box plot of percent infection reduction after IVIG according to response to polysaccharide vaccine

**Conclusion:**

Even patients with relatively mild immunoglobulin deficiencies can experience a significant reduction in recurrent infections and improved quality of life when treated with immunoglobulin. IVIG is usually well tolerated for many years but may require adjunctive steroids, IV hydration, or brand change to limit adverse side effects.

**Disclosures:**

**All Authors**: No reported disclosures.

**Figure 1:** Pre-treatment Antibody Deficit and Infection Reduction by IVIG

Plot of percent deficit of most deficient antibody subclass against infection reduction by IVIG (% of yearly pre-IVIG infections)

Infection Reduction by IVIG According to Vaccine Responsiveness

